# Case Report: Reversible central hypothyroidism-like thyroid dysfunction revealing ACTH-independent cushing syndrome during pregnancy

**DOI:** 10.3389/fmed.2026.1960906

**Published:** 2026-09-23

**Authors:** Xinyu Li, Yu Wang, Nan Gu, Wei Wang, Ying Gao

**Affiliations:** Department of Endocrinology, Peking University First Hospital, Beijing, China

**Keywords:** adrenal adenoma, adrenalectomy, Cushing syndrome, discordant thyroid function tests, pregnancy

## Abstract

Thyroid dysfunction is common during pregnancy, and a reduced free thyroxine (FT4) concentration is often attributed to gestational thyroid disease. However, low FT4 with an inappropriately non-elevated thyroid-stimulating hormone (TSH), particularly when the abnormality persists during levothyroxine treatment, should prompt reassessment and raise suspicion for central hypothyroidism rather than automatic dose escalation. We report a 29-year-old woman referred at 17 weeks of gestation because FT4 remained low (9.18 pmol/L) while TSH was 0.5 μIU/mL during levothyroxine 75 μg/day. This discordant pattern was not typical of primary hypothyroidism. Review of the broader clinical picture revealed rapid weight gain, moon face, buffalo hump, and purple abdominal striae. Cortisol evaluation demonstrated loss of diurnal rhythm, markedly elevated 24-hour urinary free cortisol, and suppressed adrenocorticotropic hormone; magnetic resonance imaging identified a right adrenal mass. ACTH-independent Cushing syndrome caused by an adrenal adenoma was diagnosed. Retroperitoneal laparoscopic adrenalectomy was performed at 18 weeks and 3 days of gestation, followed by hydrocortisone replacement. At the 1-year follow-up, free triiodothyronine (FT3), FT4, and TSH had normalized. Levothyroxine was subsequently tapered and was discontinued at the 2-year follow-up, without recurrence of hypothyroidism. The patient delivered a live-born girl at 39 weeks. This case highlights persistent discordance between FT4 and TSH during levothyroxine treatment as a sentinel clue to occult hypercortisolism in pregnancy. Early recognition can prevent diagnostic delay, avoid unnecessary long-term thyroid hormone replacement, and permit definitive treatment during the optimal gestational window.

## Introduction

Thyroid function abnormalities are frequently encountered during pregnancy. Physiological changes in thyroid-binding proteins, human chorionic gonadotropin, iodine metabolism, and plasma volume alter both TSH and FT4, making gestational- and assay-specific reference intervals essential for interpretation (1, 2). Most clinically relevant hypothyroidism in pregnancy is primary and is therefore characterized by an appropriately increased TSH. By contrast, a low FT4 concentration with a low or normal TSH is biochemically discordant and should raise consideration of central hypothyroidism, nonthyroidal illness, medication effects, including glucocorticoids, dopamine agonists, and somatostatin analogues that may suppress TSH secretion, or analytical interference rather than being assumed to represent uncomplicated primary hypothyroidism (3, 4).

Endogenous hypercortisolism is an important but easily overlooked cause of a central hypothyroidism-like pattern. Excess cortisol can inhibit hypothalamic thyrotropin-releasing hormone secretion, blunt pituitary TSH secretion and its nocturnal surge, and alter peripheral thyroid hormone metabolism. Consequently, patients with active Cushing syndrome may have low or low-normal FT4 and FT3 concentrations with an inappropriately non-elevated TSH, and these abnormalities may recover after cure of hypercortisolism (5, 6).

Cushing syndrome during pregnancy is rare because chronic cortisol excess commonly impairs ovulation and fertility. When pregnancy occurs, diagnosis is particularly difficult because weight gain, edema, hypertension, striae, and glucose intolerance overlap with normal or common pathological changes of gestation, while physiological activation of the hypothalamic-pituitary-adrenal axis complicates biochemical testing (7–9). Nevertheless, uncontrolled disease is associated with substantial maternal and fetal morbidity, and early diagnosis is critical because definitive surgery is generally most favorable in the second trimester (7–11).

Here, we describe a pregnant woman in whom persistently low FT4 accompanied by an inappropriately low/non-elevated TSH during levothyroxine treatment became the starting point for a broader endocrine reassessment and ultimately led to the diagnosis of ACTH-independent Cushing syndrome caused by a right adrenal adenoma. The diagnostic sequence and postoperative reversal of thyroid dysfunction are the central features of this report.

## Case presentation

A Chinese woman aged 29 years and 17 days, of Han ethnicity, was admitted to the Endocrinology Ward of Peking University First Hospital in Beijing, China, in July 2020 at 17 weeks of gestation because of persistent abnormal thyroid function during levothyroxine treatment. She reported normal thyroid function before pregnancy. At a routine obstetric examination at 11 weeks of gestation, thyroid function testing showed TSH 1.18 μIU/mL (reference range 0.12–3.54 μIU/mL), FT3 3.22 pmol/L (reference range 3.5–6.5 pmol/L), and FT4 6.0 pmol/L (reference range 13.33–21.97 pmol/L). She was referred to the endocrinology clinic at our hospital, initiated oral levothyroxine (LT4) at a dose of 75 μg/day. Thyroid autoantibodies were within the normal range. While taking this dose, thyroid function tests showed TSH 0.5 μIU/mL (reference range 0.78–4.25 μIU/mL), FT3 2.9 pmol/L (reference range 3.5–5.0 pmol/L), and FT4 9.18 pmol/L (reference range 11.57–16.62 pmol/L). The low FT4 concentration was accompanied by an inappropriately low TSH and was therefore not consistent with the usual biochemical pattern of primary hypothyroidism; it raised suspicion for central suppression of the hypothalamic-pituitary-thyroid axis. This biochemical discordance prompted reassessment of the original diagnosis and a search for secondary causes. Her history was also notable for rapid weight gain and menstrual irregularity before pregnancy.

Further review revealed marked hyperglycemia during pregnancy that was difficult to control despite multiple daily insulin injections. Her regimen comprised insulin aspart 12 IU before breakfast, 12 IU before lunch, and 15 IU before dinner, together with insulin detemir 25 IU at bedtime. The combination of severe insulin requirement, rapid weight gain, and atypical thyroid biochemistry raised suspicion that the metabolic and thyroid abnormalities might share an underlying endocrine cause.

On admission, her height was 1.65 m and weight was 101 kg, corresponding to a BMI of 37.1 kg/m^2^ at 17 weeks of gestation; blood pressure was 131/91 mmHg. Physical examination showed a moon face, buffalo hump, and broad purple striae over the abdomen (Figure 1). These findings strengthened suspicion of hypercortisolism. Serum cortisol at 8:00 am was 26.75 μg/dL (reference range, 4.4–19.9 μg/dL), and the normal diurnal rhythm was absent: cortisol concentrations were 26.75, 24.43, and 23.16 μg/dL at 8:00 am, 4:00 pm, and midnight, respectively. ACTH was suppressed to <1 pg/mL, and 24-hour urinary free cortisol was markedly elevated at 3,902.25 μg/24 h (reference range, 370–639 μg/24 h).

**Figure 1:**
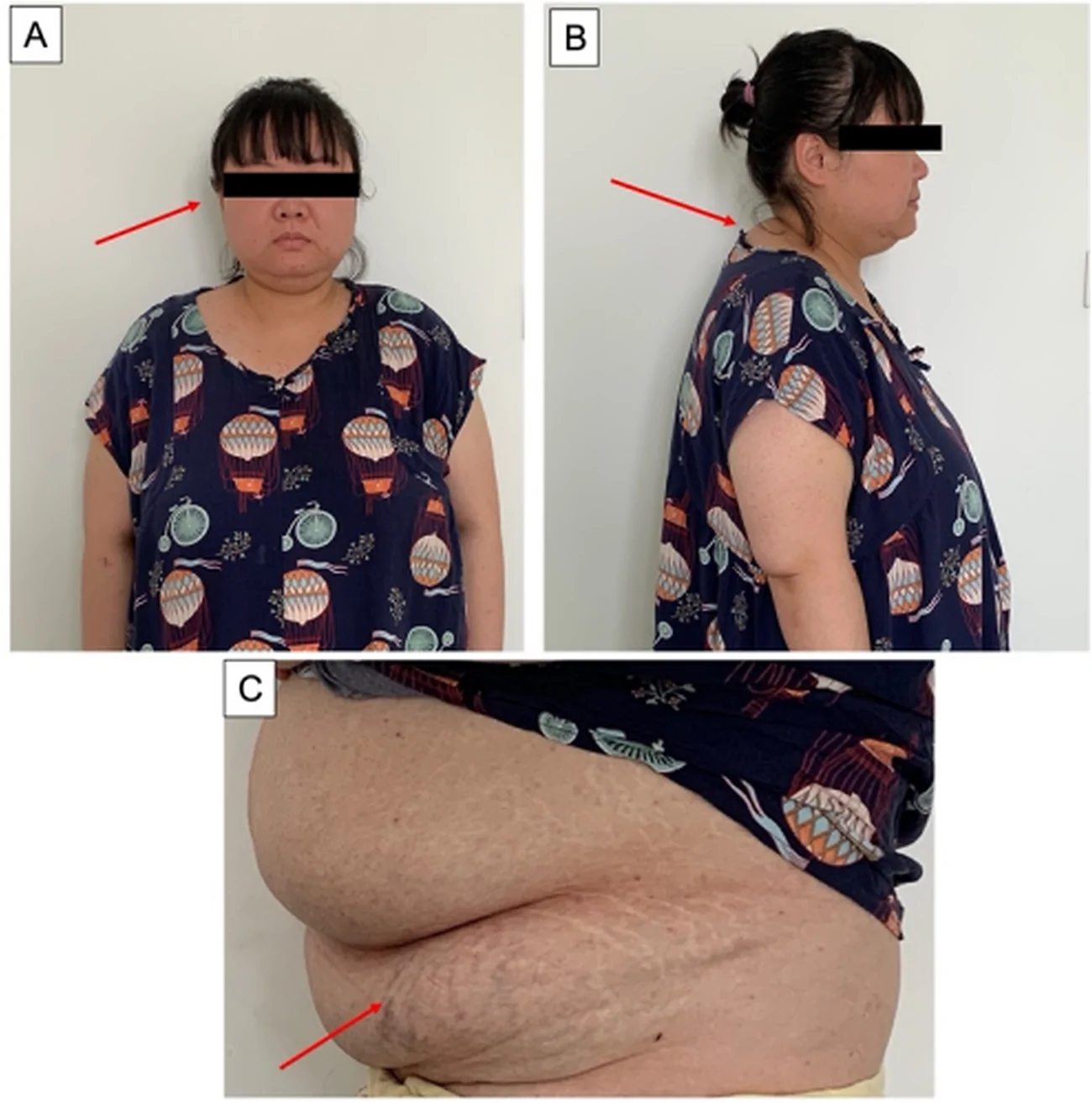
Clinical features suggestive of cushing syndrome at 17 weeks of gestation. **(A)** Moon face; **(B)** buffalo hump; **(C)** broad purple abdominal striae (red arrows).

Non-contrast adrenal magnetic resonance imaging demonstrated a 3.9 × 2.5 × 2.1 cm right adrenal mass (Figures 2A,B). Suppressed ACTH together with markedly elevated 24-hour urinary free cortisol supported ACTH-independent Cushing syndrome, and the right adrenal lesion was considered the likely source of cortisol excess.

**Figure 2:**
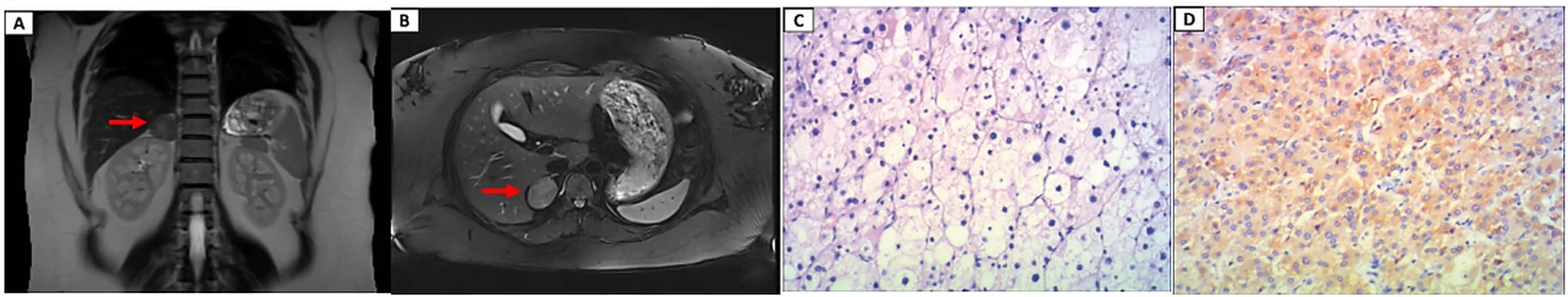
Adrenal imaging and histopathological findings. **(A,B)** Non-contrast upper abdominal magnetic resonance imaging showed a 3.9 × 2.5 × 2.1 cm right adrenal mass (red arrows). **(C)** Hematoxylin and eosin staining of the right adrenal cortical adenoma (×200). **(D)** Inhibin immunohistochemical staining of the right adrenal cortical adenoma (×200).

After multidisciplinary discussion among endocrinology, anesthesiology, urology, obstetrics, and gynecology teams, retroperitoneal laparoscopic resection of the right adrenal lesion was performed at 18 weeks and 3 days of gestation. Hydrocortisone replacement was initiated perioperatively. During adrenalectomy, 200 mg of intravenous hydrocortisone was administered, followed by an additional 100 mg intravenously later on the day of surgery. Postoperatively, hydrocortisone was tapered from 100 mg every 8 h for 1 day to 50 mg every 6 h for 2 days, 50 mg every 8 h for 1 day, and 50 mg every 12 h for 1 day, followed by oral hydrocortisone 80 mg/40 mg (morning/afternoon). Histopathological examination confirmed an adrenal cortical adenoma (Figures 2C,D). Glycemic control improved after adrenalectomy; postoperative HbA1c was 6.1%. FT3 and FT4 normalized early, whereas TSH remained suppressed, likely reflecting ongoing levothyroxine treatment, glucocorticoid-induced TSH suppression, and gradual recovery of the hypothalamic-pituitary-thyroid axis.

The ACTH-adrenal axis recovered gradually, as shown in Table 1. ACTH was detectable but remained suppressed at 1.28 pg/mL 2 weeks after surgery and throughout pregnancy. Hydrocortisone was adjusted to 60/20 mg (morning/evening) at 23 weeks of gestation and to 30/20/20 mg (morning/afternoon/evening) at 25 weeks. At 39 weeks of gestation, the patient delivered a live-born girl by cesarean section. Peripartum hydrocortisone was administered intravenously as detailed in Table 1. Oral hydrocortisone was resumed on postpartum day 3 and tapered off by 1 year and 4 months postpartum. The newborn had low birth weight but subsequently showed normal growth and development. Thyroid function normalized by the 1-year follow-up, and levothyroxine was discontinued at the 2-year postoperative follow-up without recurrence of hypothyroidism.

**Table 1:** Longitudinal endocrine and metabolic findings before and after adrenalectomy.

Time point	FT3 (pmol/L)	FT4 (pmol/L)	TSH (μIU/mL)	ACTH (pg/mL)	Cortisol (μg/dL)	LT4 dose (μg/day)	Hydrocortisone regimen
11 weeks of gestation	3.22 (3.5–6.5)	6 (13.33–21.97)	1.18 (0.12–3.54)	-	-	Initial 75	-
13 weeks of gestation	2.9 (3.5–6.5)	9.18 (11.57–16.62)	0.5 (0.78–4.25)	<1 (7.2–63.3)	08:00 27.26 (4.4–19.9)	75	-
17 weeks of gestation	3.42 (3.5–6.5)	18.36 (11.57–16.62)	0.02 (0.78–4.25)	<1 (7.2–63.3)	08:00 26.75, 16:00 24.43, midnight 23.16	75	-
Adrenalectomy (18 weeks and 3 days of gestation) and early postoperative period	-	-	-	-	-	75	Day of surgery: 200 mg IV intraoperatively + 100 mg IV postoperatively (300 mg total) POD 1: 100 mg IV q8 h (300 mg/day) POD 2–3: 50 mg IV q6 h (200 mg/day) POD 4: 50 mg IV q8h (150 mg/day) POD 5: 50 mg IV q12 h (100 mg/day) Thereafter: 80 mg/40 mg PO (morning/afternoon; 120 mg/day) followed by gradual tapering
23 weeks of gestation	4.29 (3.5–6.5)	20.74 (11.57–16.62)	0.07 (0.78–4.25)	1.4 (7.2–63.3)	08:00 0.91 (4.4–19.9)	75 (Mon→Fri) and 50 (Sat→Sun)	60 mg/20 mg (morning/evening; 80 mg/day)
25 weeks of gestation	4.78 (3.5–6.5)	12.08 (11.57–16.62)	0.06 (0.78–4.25)	1.46 (7.2–63.3)	08:00 8.57 (4.4–19.9)	75 (Mon→Fri) and 50 (Sat→Sun)	30 mg/20 mg/20 mg (morning/afternoon/evening; 70 mg/day)
Delivery and early postpartum period (39 weeks of gestation)	-	-	-	-	-	75 (Mon→Fri) and 50 (Sat→Sun)	Delivery day: 100 mg IV q12 h (200 mg/day) PPD 1: 50 mg IV q12 h (100 mg/day) PPD 2: 25 mg IV q12 h (50 mg/day) PPD 3 onward: 30 mg/20 mg/20 mg PO (morning/afternoon/evening; 70 mg/day), followed by gradual tapering
1 year 4 months after delivery	5.44 (3.5–6.5)	15.17 (11.48–22.70)	4.14 (0.55–4.78)	90.47 (7.2–63.3)	08:00 18.84 (4.4–19.9)	25	discontinued
2-year postoperative follow-up	5.16 (3.5–6.5)	13.52 (11.48–22.70)	2.44 (0.55–4.78)	115 (7.2–63.3)	08:00 7.9 (4.4–19.9)	discontinued	

Values in parentheses indicate the corresponding reference ranges. LT4, levothyroxine; FT3, free triiodothyronine; FT4, free thyroxine; TSH, thyroid-stimulating hormone; ACTH, adrenocorticotropic hormone; IV, intravenous; PO, oral; POD, postoperative day; PPD, postpartum day; q6 h, every 6 h; q8 h, every 8 h; q12 h, every 12 h; Mon→Fri, Monday through Friday; Sat→Sun, Saturday through Sunday; -, data not available. For oral hydrocortisone, doses separated by slashes indicate sequential doses administered at the times of day specified in parentheses. Hydrocortisone doses are reported as administered doses, with the total daily dose shown where applicable.

## Discussion

This case highlights that persistent discordance between FT4 and TSH during levothyroxine treatment should prompt etiological reassessment. In primary hypothyroidism, a low FT4 should ordinarily provoke an increase in TSH; inadequate levothyroxine replacement in pregnancy likewise usually presents with a persistently elevated or rising TSH. Therefore, persistently low FT4 with non-elevated or suppressed TSH should prompt confirmation and etiological reassessment rather than levothyroxine escalation alone (1–4).

Interpreting this pattern during pregnancy requires particular caution. Thyroid function tests are physiologically changed by gestational changes, and trimester- and assay-specific reference intervals should be used whenever available (1, 2). The differential diagnosis of decreased FT4 includes isolated maternal hypothyroxinemia, assay interference, LT4 treatment, nonthyroidal illness, and central hypothyroidism (3, 4). In our patient, the abnormal TSH-FT4 relationship, rather than the isolated FT4 value, indicated that replacement therapy alone could not explain the biochemical picture. Nevertheless, continuing levothyroxine while the underlying cause was being investigated was considered a cautious interim strategy, because adequate maternal T4 availability is important for fetal development, particularly fetal brain development, and under-replacement should be avoided during pregnancy (1, 3).

The subsequent clinical and biochemical findings provided a mechanistic explanation. Glucocorticoid excess suppresses the hypothalamic-pituitary-thyroid axis by reducing TRH drive, TSH pulse amplitude, and the nocturnal TSH surge, while also inhibiting peripheral conversion of T4 to T3 and reducing thyroid hormone-binding proteins (5, 6). In a cohort of patients with active Cushing syndrome, Shekhar et al. found a central hypothyroidism-like pattern in 53%, and thyroid hormone concentrations generally recovered over 6–12 months after curative surgery ^(^5). This delayed recovery closely parallels our patient's course: FT3 and FT4 improved early after adrenalectomy, whereas complete normalization of the axis and successful withdrawal of levothyroxine required longer follow-up.

Pregnancy can obscure Cushing syndrome because gestational changes overlap with hypercortisolism. In this patient, loss of diurnal cortisol variation, markedly elevated urinary free cortisol, repeatedly suppressed ACTH, and a unilateral adrenal mass formed a coherent biochemical and anatomical diagnosis of adrenal Cushing syndrome (7–9). Severe insulin resistance was a corroborating clue, whereas the low-FT4/low-TSH pattern triggered the broader endocrine reassessment.

A recent report also described central hypothyroidism in a pregnant patient with adrenal Cushing syndrome and withdrawal of thyroid hormone after treatment, confirming that this association can occur but remains rarely emphasized in pregnancy literature (12). Our case extends that observation by making persistent TSH-FT4 discordance during levothyroxine therapy the explicit diagnostic entry point and by documenting the temporal separation between rapid metabolic improvement and delayed thyroid-axis recovery. These features distinguish the case from the more typical pregnancy Cushing reports centered on hypertension, hypokalemia, or gestational diabetes (12, 13).

Early recognition is important because active Cushing syndrome increases maternal and fetal risk, including hypertensive disorders, diabetes, preterm birth, fetal growth impairment, and fetal loss (10, 11). Delayed diagnosis can be severe, as illustrated by a reported case with intrauterine fetal death (14). For a localized adrenal source, laparoscopic adrenalectomy in the second trimester is generally preferred when disease severity and maternal-fetal status support intervention (8, 9, 15, 16). In the present case, identification at 17 weeks allowed surgery at 18 weeks and 3 days, before progressive metabolic complications could further compromise the pregnancy.

This case highlights three clinically relevant points. First, thyroid dysfunction was part of the diagnostic presentation rather than an incidental finding after Cushing syndrome was established. Second, postoperative improvement in glycemic control and thyroid function supports hypercortisolism as a common and reversible driver of both abnormalities. Finally, sustained thyroid recovery at 1 year underscores the importance of reassessing levothyroxine after correction of hypercortisolism rather than assuming lifelong replacement.

In conclusion, hypothyroidism is common in pregnancy, but a low FT4 concentration with an inappropriately non-elevated TSH that persists during levothyroxine treatment is not a routine primary hypothyroid pattern. This discordance may be a sentinel manifestation of Cushing syndrome. Recognizing the pattern early, integrating it with Cushingoid features, and pursuing timely cortisol evaluation can prevent missed diagnosis, preserve the optimal surgical window, and avoid unnecessary long-term thyroid hormone replacement.

## Patient perspective

I underwent adrenal surgery during pregnancy after Cushing syndrome was diagnosed. My glucose and thyroid function gradually improved after treatment, and I delivered my daughter at 39 weeks. During follow-up, both hydrocortisone and levothyroxine were eventually discontinued. I was satisfied with the treatment outcome and the subsequent recovery.

## Data Availability

The raw data supporting the conclusions of this article will be made available by the authors, without undue reservation.
